# Control of Morphology and Substrate Etching in InAs/InP Droplet Epitaxy Quantum Dots for Single and Entangled Photon Emitters

**DOI:** 10.1021/acsanm.2c01197

**Published:** 2022-05-30

**Authors:** Raja Sekhar Reddy Gajjela, Elisa Maddalena Sala, Jon Heffernan, Paul M. Koenraad

**Affiliations:** † Department of Applied Physics, Eindhoven University of Technology, Eindhoven 5612 AZ, The Netherlands; ‡ EPSRC National Epitaxy Facility, The University of Sheffield, North Campus, Broad Lane, S3 7HQ Sheffield, United Kingdom; § Department of Electronic and Electrical Engineering, The University of Sheffield, Sir Frederick Mappin Building, Mappin Street, S1 3JD Sheffield, United Kingdom

**Keywords:** InAs/InP quantum dots, droplet epitaxy, substrate etching, X-STM, InAs etch pits

## Abstract

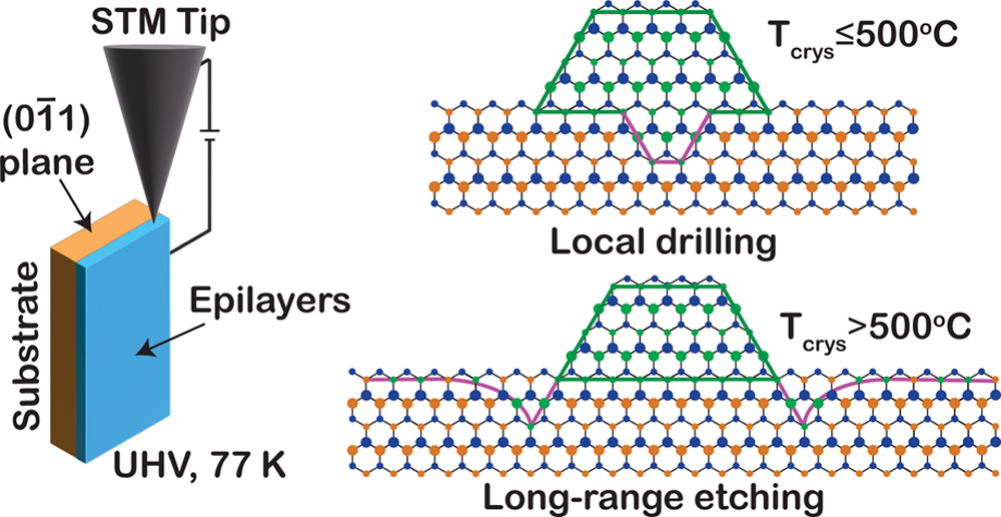

We present a detailed atomic-resolution study of morphology and substrate etching mechanism in InAs/InP droplet epitaxy quantum dots (QDs) grown by metal–organic vapor phase epitaxy via cross-sectional scanning tunneling microscopy (X-STM). Two different etching processes are observed depending on the crystallization temperature: local drilling and long-range etching. In local drilling occurring at temperatures of ≤500 °C, the In droplet locally liquefies the InP underneath and the P atoms can easily diffuse out of the droplet to the edges. During crystallization, the As atoms diffuse into the droplet and crystallize at the solid–liquid interface, forming an InAs etch pit underneath the QD. In long-range etching, occurring at higher temperatures of >500 °C, the InP layer is destabilized and the In atoms from the surroundings migrate toward the droplet. The P atoms can easily escape from the surface into the vacuum, forming trenches around the QD. We show for the first time the formation of trenches and long-range etching in InAs/InP QDs with atomic resolution. Both etching processes can be suppressed by growing a thin layer of InGaAs prior to the droplet deposition. The QD composition is estimated by finite element modeling in combination with X-STM. The change in the morphology of QDs due to etching can strongly influence the fine structure splitting. Therefore, the current atomic-resolution study sheds light on the morphology and etching behavior as a function of crystallization temperature and provides a valuable insight into the formation of InAs/InP droplet epitaxy QDs which have potential applications in quantum information technologies.

## 1 Introduction

Semiconductor quantum dots (QDs) have been extensively studied in the past two decades and optimized for a wide range of applications such as QD lasers,^1−3^ photovoltaics,^4,5^ single photon emitters,^6−10^ flash memories,^11,12^ and quantum communication and information technologies.^13−15^ The well-established Stranski–Krastanov (SK) growth of III–V semiconductor QDs has certain limitations such as the requirement for lattice-mismatched materials, limited control over QD growth, QDs sitting on top of a two-dimensional (2D) wetting layer (WL), and extension of QDs in one of the crystal directions. Droplet epitaxy (DE) developed by Koguchi et al.,^16^ where group III and V fluxes are introduced separately into the growth chamber, offers more degrees of freedom than SK growth to control the QD growth. In DE, controlling the droplet formation offers precise tuning of QD size and areal density. The final shape of the QDs strongly depends on the surface reconstruction, the group V flux, and the crystallization temperature.^17,18^ Moreover, metal–organic vapor phase epitaxy (MOVPE) growth of such QDs enables the large-scale fabrication of high-quality QDs for applications in the telecom C-band.

InAs/InP self-assembled QDs grown via DE by MOVPE emitting at ∼1.55 μm have shown 4 times lower fine structure splitting (FSS) and superior coherence in emission compared to SK grown InAs/InP QDs.^19,20^ DE can also be used to establish local droplet etching (LDE) of the substrate by the metal droplets which can subsequently be used to create preferential nucleation sites for the QDs.^21,22^ Growing InAs/InP in LDE mode offers more advantages in obtaining QDs with reduced shape asymmetry and smaller FSS.^23,24^ LDE of GaAs/AlGaAs QDs has been extensively studied where the surface etching is performed with aluminum droplets and the etched holes are filled with GaAs followed by AlGaAs capping.^25−27^ Recently, Sala et al.^22^ has shown the formation of InAs/InP DEQDs in locally etched pits depending on the crystallization temperature, where the QDs appeared to sit at the center of the etched region. However, the research on the droplet etching mechanism in InAs/InP QDs is limited, and understanding the droplet etching is essential for further optimization of the QD growth. In this work, we address the formation of QDs and the etching mechanism as a function of crystallization temperature with atomic resolution.

Cross-sectional scanning tunneling microscopy (X-STM) is a unique technique that can provide detailed atomic-scale characterization of the embedded QDs, revealing alloying around the QDs due to QDs apex leveling, alloy intermixing in the QDs, segregation, etc.^28−33^ In a previous article, we showed for the first time the presence of localized InAs etch pits in InAs/InP DEQDs with atomic resolution, where the etch pits are formed due to the local liquefaction of the substrate by the In droplet, followed by crystallization in the As-rich environment.^34^ In this article, we study the mechanism of substrate etching when tuning the crystallization temperature of the indium droplets with atomic resolution by X-STM. We present a detailed morphological and compositional analysis of the DEQDs crystallized at 480 and 520 °C. We observed two types of etching processes as a function of crystallization temperature, which we name “local drilling” and “long-range etching”. Local drilling of the In droplet into the InP substrate occurs at lower crystallization temperatures, i.e., 480 °C. At higher temperatures of >500 °C, after the droplet deposition the whole surface destabilizes and the In droplet acts as a sink pulling indium from the sides, thus forming trenches on all sides of the QDs. The etching around the QD appeared to increase with the increasing crystallization temperature.^22^ Here, we discuss the mechanism of such trench formation for the first time with atomic resolution. We show that both etching processes can be suppressed by growing a thin InGaAs interlayer prior to the droplet deposition as suggested by Sala et al.^35^ QDs crystallized at 520 °C have lower size inhomogeneity compared to QDs crystallized at 480 °C. We note that, in our previous work,^34^ the indium droplets were formed at 400 °C and the crystallization temperature was up to 500 °C. In this work, we modified the growth conditions to assess the effect of growth conditions on QD morphology and substrate etching. Therefore, the current work is one step forward in the growth optimization process.

## 2 Results and Discussion

We measured two samples with InAs/InP DEQDs, where sample A had two layers of QDs crystallized at 480 (layer A1) and 520 °C (layer A2). In sample B, the QDs were grown on top of a 5 nm InGaAs layer instead of InP and crystallized at 520 °C. Additional measurements are performed on samples similar to sample B by changing the crystallization temperature to 480 °C and the thickness of the InGaAs layer to 1 nm. A detailed growth description can be found in section 4.

### 2.1 Morphology of the QDs

**Figure 1:**
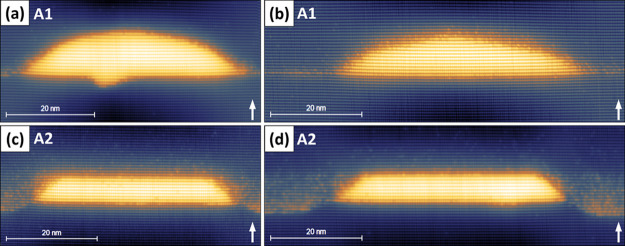
X-STM filled-state topographic images of InAs/InP DEQDs in layer A1 (a, b) and layer A2 (c, d) taken at a bias voltage (*V*_b_) = −3.0 V and tunnel current (*I*_t_) = 50 pA. The dark to bright contrast indicates an outward relaxation (∼0.25–0.30 nm) of the InAs QDs. The growth direction [100] is indicated by the white arrow.

In Figure 1, we present topographic filled-state X-STM images of DEQDs in both layers A1 (parts a and b) and A2 (parts c and d), measured with tunnel conditions: a sample bias voltage (*V*_b_) of −3.0 V and a tunnel current set point (*I*_t_) of 50 pA. The relative height of the X-STM tip from the cleaved surface generates the contrast in the images. The InAs QDs which are compressively strained with InP relax outward immediately upon cleaving, and this outward relaxation strongly depends on QD size and composition. In our case, the dark to bright contrast represents a height difference of ∼0.25–0.30 nm. The electronic contribution to the X-STM images was suppressed by performing filled-state imaging at a higher negative bias voltage (sample bias with respect to the tip) obtaining a pure structural contrast.^36,37^ In Figure 1a,b, one can notice the near dome shape of the QDs in layer A1 (parts a and b) with heights of 9.7 ± 0.5 nm for Figure 1a and 8.2 ± 0.5 nm for Figure 1b with base lengths of 50.28 ± 0.8 nm for Figure 1a and 55.00 ± 0.8 nm for Figure 1b. On the other hand, the QDs in layer A2 (Figure 1c,d) have a perfect trapezoidal shape in the cross section (truncated pyramid in 3D) with heights of 6.1 ± 0.5 nm for Figure 1c and 6.4 ± 0.5 nm for Figure 1d with base lengths of 43.28 ± 0.8 nm for Figure 1c and 51.20 ± 0.8 nm for Figure 1d. It is important to point out that all the QDs in layer A1 observed in the X-STM measurement are very similar in shape to the QDs shown in Figure 1a,b and all the QDs in layer A2 are almost identical to the QDs shown in Figure 1c,d in both size and shape. AFM analysis of QDs grown under identical growth conditions^22^ showed that the QDs have similar sizes in both directions ([110] and [11̅0]), compared to standard InAs/InP SKQDs^19^ which usually extend in one of the {110} crystal directions. The symmetry in QD size and shape is crucial for optoelectronic applications as it strongly affects the QD FSS.

**Figure 2:**
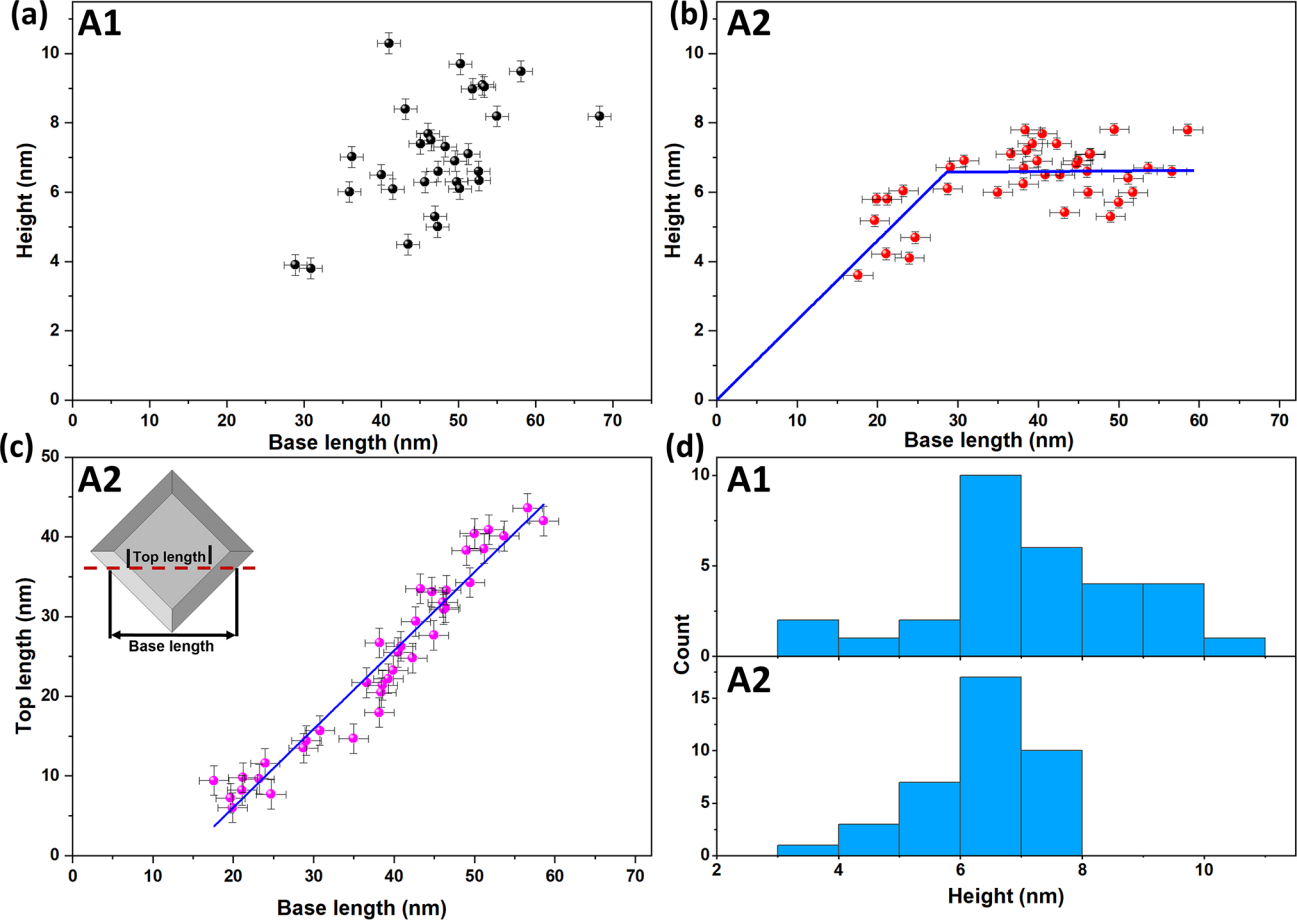
Height versus base length of DEQDs in layer A1 (a) and layer A2 (b) and top length versus base length of DEQDs in layer A2 (c) measured from the filled-state X-STM images with a linear fit in blue. The most likely shape of the DEQDs in layer A2 is shown at the top left corner of (c) with a cleaving plane in red. The height distribution of DEQDs in layers A1 and A2 is given in (d).

It is obvious that we observe the biggest QD when the cleaving plane runs through the middle of the QD. By default, the cleaving process is completely random, meaning that the cleaving plane does not run through the middle of each and every QD. Hence, we observe different heights and base lengths in the cross-section images based on cleaving position and we can utilize this data to estimate the 3D morphology of the QD with the help of a geometrical model first proposed by Bruls et al.^30,38^ In Figure 2, we provide the height versus base length data of all 30 QDs in layer A1 (Figure 2a) and 38 QDs in layer A2 (Figure 2b) measured from all X-STM images. The height versus base length data of QDs in layer A1 (Figure 2a) appear to be scattered, and it is difficult to extract any reliable arguments about the 3D shape and size of the QDs. The scattering of the data could be due to the shape of the QD as it is close to a dome rather than a trapezium. On the other hand, there is a clear trend for QDs in layer A2: a linear increase in height with base length and the height saturated at 6.5 ± 1.0 nm as shown in Figure 2b. This relation corresponds very well with model 2 (square-based truncated pyramid QD) from Bruls et al.,^30,38^ meaning that the cleaving plane is parallel to the diagonal of the near square-based pyramid. Therefore, the base length reported in Figure 2b is  times higher than the actual base length. In Figure 2c, we observe a clear linear dependence of top length as a function of base length of the QDs in layer A2, providing additional confirmation that the cleaving plane is parallel to the diagonal of the square-based QD as shown in the top left corner of Figure 2c. The density of QDs is lower in layer A1 than in A2, and also the size inhomogeneity is higher in A1 than in A2. The height distribution of QDs in both layers A1 and A2 is shown in Figure 2d; the QDs in layer A1 have a larger distribution of heights than the QDs in layer A2. This provides additional confirmation that the QDs in layer A2 are more size homogeneous, especially in height, than the QDs in layer 1.

The DEQDs in layer A1 (Figure 1a,b) appear to be near dome shaped with a small top facet. The slope of the side facet changed with the increasing height of the QD. On the other hand, QDs in layer A2 (Figure 1c,d) are perfectly trapezium-shaped (truncated pyramids in 3D) with a flat and long top facet and reduced height compared to QDs in layer A1. This is due to the fact that QDs in layer A2 were capped at higher temperature (520 °C), and it is well-known that the high-temperature capping induces the leveling of QDs due to the increased mass transfer from the QD apex to the sides.^39−41^ Two distinct features can be observed in Figure 1: (1) a small etch pit of nearly pure InAs is visible underneath the QD in Figure 1a; (2) the QDs in layer A2 (Figure 1c,d) appeared to be elevated compared to the wetting layer due to the formation of trenches around the QDs. The two features are a direct result of the change in the substrate etching mechanism due to the increased crystallization temperature from layer A1 (480 °C) to layer A2 (520 °C) and are explained in detail in section 2.2.

### 2.2 Etching Mechanism

The droplet etching of the substrate is a complex phenomenon mainly involving dissolution and mass transport of the substrate chemical species (In and P atoms). The droplet etching was initially observed during the growth of GaAs/AlGaAs DEQDs in molecular beam epitaxy (MBE), where the local etch pit formation (local etching) was studied thoroughly.^21,25,26,42,43^ However, the research on In droplet etching in InP and in MOVPE is very limited, and in our previous work,^34^ we showed the presence of localized InAs etch pits underneath the QDs for the first time in InAs/InP DEQDs with atomic resolution. The etch pit formation strongly depends on growth conditions and reaction kinetics during the droplet formation and crystallization. As mentioned before, two different etching mechanisms are observed in InAs/InP droplet epitaxy as a function of crystallization temperature, which we name as local drilling for crystallization temperatures of ≤500 °C occurring underneath the droplet and long-range etching around the droplet due to the surface destabilization of InP for crystallization temperatures of >500 °C. From AFM analysis it appeared that the etching around the dot increases with the increasing crystallization temperature due to the increased atomic migration and desorption.^22^

**Figure 3:**
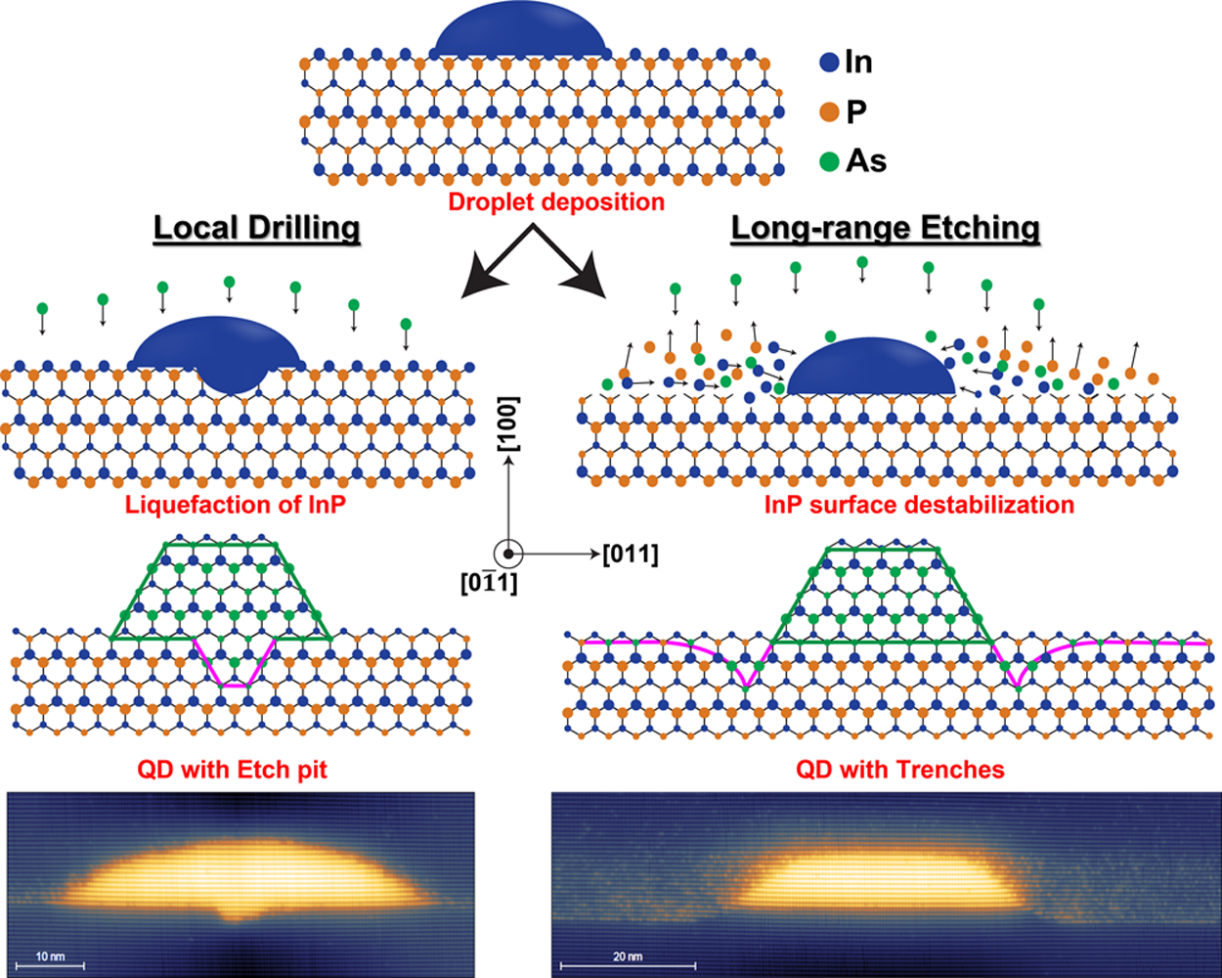
Schematic explanations of two different etching processes: local drilling and long-range etching. Initially In droplets are formed on the InP surface. In local drilling, the etching starts with local liquefaction of InP underneath the droplet followed by etch pit formation during the crystallization. In long-range etching, the InP surface is destabilized at high temperature and the trenches are formed due to the In migration toward the droplet, while the P atoms escape back into the chamber as shown in the figure. The blue circles are In atoms, the orange circles are P atoms, the green circles are As atoms, and the pink line represents the etching. X-STM filled-state topographic images of InAs/InP DEQDs taken at *V*_b_ = −3.0 V and *I*_t_ = 50 pA shown for layer A1 with a local etch pit and for layer A2 with trenches around the QD.

**Figure 4:**
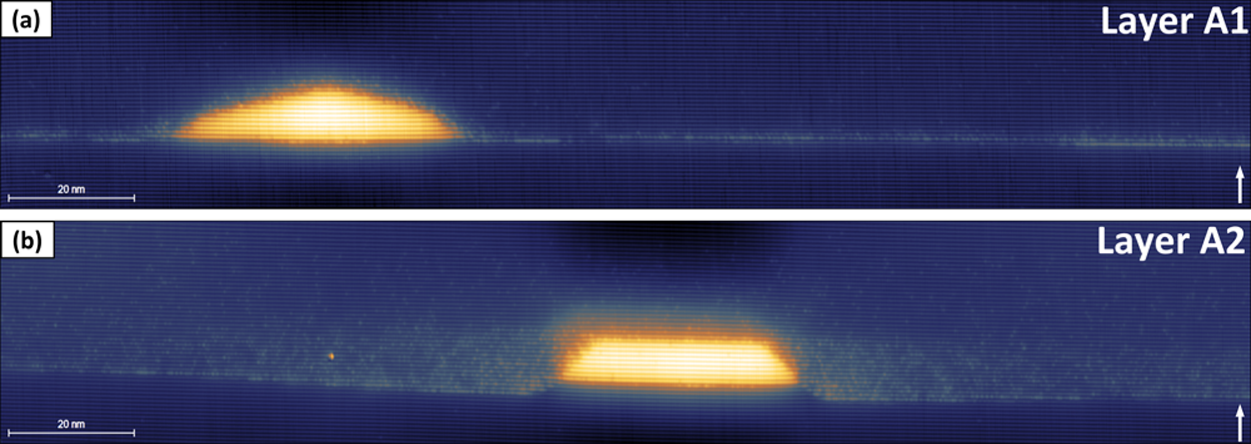
X-STM filled-state topographic images of InAs/InP DEQDs taken at *V*_b_ = −3.0 V and *I*_t_ = 50 pA. The 200 nm long images of both layer A1 (a) and layer A2 (b) clearly shows the effect of long-range etching. The growth direction [100] is indicated by the white arrow.

The two etching mechanisms are schematically shown in Figure 3 along with X-STM filled-state topographic images. In both cases, the process starts with an In droplet deposition on the InP layer. Local drilling is dominant for temperatures of ≤500 °C; below this growth temperature both the InP and liquid In are stable phases.^44^ The local liquefaction of InP below the In droplet, dissolution, and the solid solubility of individual elements determine the extent of droplet etching also known as local drilling of the substrate. The P atoms can easily diffuse out of the droplet, and subsequently, during the crystallization, the As atoms diffuse into the droplet crystallizing at the liquid–solid interface and leading to the formation of a nearly pure InAs etch pit as shown schematically and with atomic resolution in Figure 3. In the current study, we found that the etch pit size is nearly uniform everywhere ∼3 bilayers (BLs) deep into the substrate and almost two-thirds of the QDs found during the X-STM experiment are without a localized etch pit as shown in the X-STM image in Figure 4a. In our previous work, we presented a detailed atomic-scale study on the formation of InAs etch pits;^34^ we observed varied etch pit sizes arbitrarily positioned underneath almost every single QD observed in X-STM.

Sala et al.^22^ published the AFM analysis of identical QDs and revealed the formation of a crater around the QDs at higher temperatures. The size of this crater increased with increasing crystallization temperature. The growth surface is In-rich, and it is likely that, at high temperatures (>500 °C), the InP becomes unstable and dissociates into elemental In and P atoms. During the crystallization of the QDs, multiple processes are taking place at once, such as destabilization of InP, As–P surface exchange, strain-driven migration of indium toward the droplet, and phosphorus atoms escaping back into the chamber^45−48^ as shown in Figure 3. From AFM analysis, it appeared that the size of the droplet increased with the crystallization temperature as the In atoms from all sides of the droplet migrate toward the droplet, increasing the total In content.^22^ The droplet size decreased for temperatures above 520 °C, suggesting the desorption of In from the growth surface back into the chamber leading to smaller droplets.^22^ The surface destabilization and the elemental migration are the major processes driving the formation of trenches around the QD as shown schematically and with atomic resolution in Figure 3.

The long-range etching is clearly visible in Figure 4b with the QD base exactly in line with the 2D quasi-WL far from the QD, and the trenches around the QD are almost 4–5 BLs deep into the underlying InP. A similar etching profile was reported by Holewa et al.^49^ for QDs crystallized at 550 °C due to the In diffusion from the droplet neighborhood toward the droplet. Holewa et al.^49^ reported a concave base for the QDs based on TEM analysis, but we show an atomically flat base for our QDs as shown in Figure 4b. The formation of these trenches was first predicted by Yoon et al.,^46^ and in this work, we proved the formation of such trenches which can be controlled by the crystallization temperature with atomic resolution X-STM images. The crystallization time for QDs in both layers A1 and A2 is constant, meaning that the temperature ramp rate is higher for layer A2 compared to layer A1. In addition to trench formation, we found that a few QDs in layer A2 have a trace of localized etch pit with a thickness of maximum 1 BL. The temperature ramp rate and indium mobility are the deciding factors for the etching mechanism. At a higher temperature ramp rate the local liquefaction is greatly suppressed. In general, it is well-established that 30–40 nm spacer thickness is enough to decouple two InAs QD layers grown on top of each other on a GaAs substrate (7.1% lattice mismatch).^50^ In the X-STM experiments, there is no indication of any strain effect of layer A1 on layer A2 as the two layers are separated by an 80 nm thick InP layer. Also, the total strain in the system (InAs/InP) is lower due to the reduced lower lattice mismatch of 3% compared to InAs/GaAs, where the lattice mismatch is 7.1%. On top of that, we have additional confirmation from finite element (FE) simulations on InAs/InP QDs where the two QD layers are separated by 80 nm thick InP and we observed no strain influence of the bottom layer on the top one. Therefore, we can confidently assert that the changes in the QD morphology and etching mechanism are totally dependent on the crystallization temperature.

**Figure 5:**
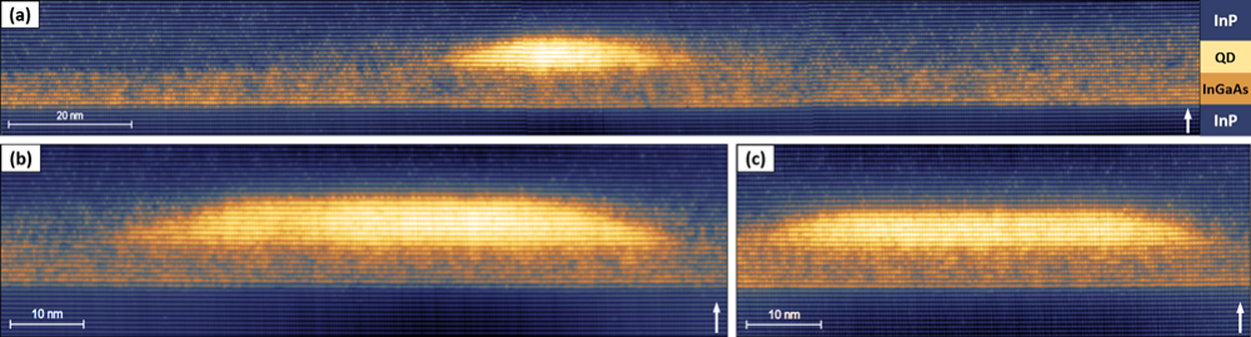
X-STM filled-state topographic images of InAs/InP DEQDs with a 5 nm InGaAs interlayer taken at *V*_b_ = −3.0 V and *I*_t_ = 50 pA. Both the local drilling and long-range etching effects are suppressed by introducing an InGaAs layer before droplet deposition. The 200 nm long image in (a) shows that the long-range etching and trench formation is absent. High-resolution images in (b) and (c) show the absence of localized etch pit and also contrast fluctuations within the QD. The growth direction [100] is indicated by the white arrow.

We also investigated a method to suppress both etching processes, local drilling and long-range etching, by growing a thin InGaAs layer on InP prior to the droplet deposition. The major driving forces for the droplet etching are surface liquefaction, destabilization, and elemental migration. Thus, by forming droplets on a thin InGaAs layer, we can suppress all of these effects. The surface As–P exchange strongly influences the QD morphology, and in the past, a lattice-matched InGaAs layer was used mainly to suppress the surface As–P exchange.^35,46,47,51^ The filled-state topographic images in Figure 5 show the size and shape of InAs DEQDs formed on top of a 5 nm InGaAs layer. The DEQDs are truncated pyramid shaped with observable contrast fluctuations within the QD. We note that the QDs grown on InP (Figure 1) have uniform contrast with minor intermixing close to the edges. Therefore, the QDs grown on InGaAs are not as pure as the ones grown on InP. We studied many topographic images, and we conclude that the local drilling effect is absent in all the DEQDs in sample B. From the 200 nm long image shown in Figure 5a, it is evident that the long-range-etching effect shown in Figure 4b is also suppressed, as expected from previous AFM and TEM investigations.^35^ Yoon et al.^46^ suggested that this is a result of the change in the growth kinetics from the mass-transport regime on InP to the surface-reaction-limited regime on InGaAs. We analyzed the QDs grown on InGaAs, crystallized at both 480 and 520 °C, and observed that both etching effects are absent. We performed additional experiments by changing the thickness of the InGaAs layer from 5 to 1 nm and observed the same result. These additional X-STM images are shown in section SII of the Supporting Information. Thus, we prove that using a thin InGaAs layer suppresses both etching processes, i.e., local drilling and long-range etching.

### 2.3 Composition of the QDs

**Figure 6:**
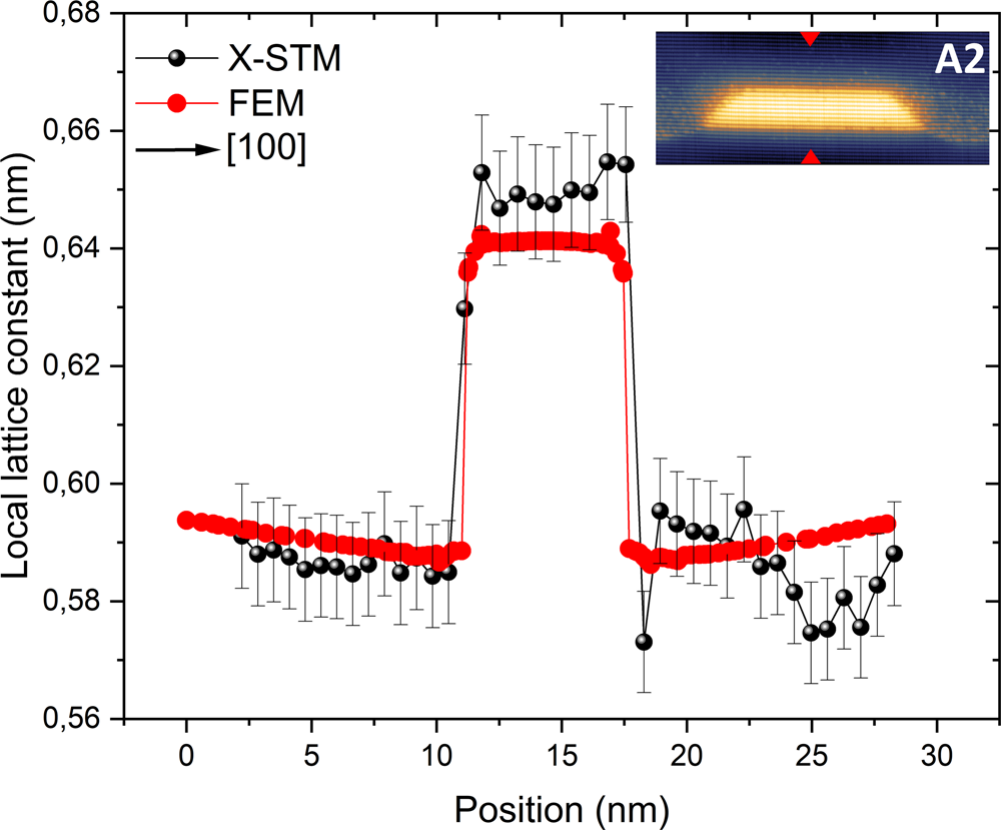
Local lattice constant profile (a) of the cleaved QD (inset X-STM image with red arrowheads indicating the position where the profile was taken) from layer A2 along the growth direction [100]. Experimental profiles measured from X-STM images are given in black, and the calculated profiles from FE simulations are given in red. The arrow indicates the growth direction [100].

The compositional fluctuations within the QDs result in a nonuniform contrast in X-STM images as the STM tip is sensitive to the local compositional changes.^36^ In the X-STM filled-state images (Figure 1), we observe a uniform contrast throughout the QDs, suggesting that the QDs are pure InAs in composition. Minor alloy fluctuations due to P intermixing can be seen close to the QD edges. Holewa et al.^49^ suggested a composition of InAs_0.8_P_0.2_ for the DEQDs crystallized and annealed at 550 °C based on TEM, energy dispersive X-ray spectroscopy (EDX), and photoluminescence measurements. However, our DEQDs crystallized at both 480 and 520 °C have pure InAs without any trace of P atoms within the QDs. This difference can be explained by the fact that at temperatures higher than 540 °C the As substitution for P atoms is higher,^52^ thus leading to greater As–P exchange reactions during the crystallization step and increased incorporation of P atoms into the QDs studied in Holewa et al.^49^ The compositional uniformity can be seen more clearly in the X-STM current images (shown in section SI of the Supporting Information) of the QDs shown in Figure 1. It is easy to identify alloy fluctuations in the current images due to the abrupt change in the current response of the STM tip due to compositional fluctuations and also the suppressed topographic contrast, meaning that a pure QD (e.g., InAs) has uniform contrast in the current image. In addition, we performed FE simulations to calculate the local lattice constant of the cleaved QD and compared them with experimental values obtained from X-STM to provide an approximate composition of the QDs. A reliable 3D model was constructed based on the structural analysis performed by X-STM for layer A2. As mentioned before, the height versus base length data of QDs in layer A1 is scattered and it is difficult to construct a reliable model for FE simulation. Figure 6 shows the local lattice constant profile of a cleaved QD from layer A2; the X-STM experimental profile is given in black and the calculated profile from the FE simulation is given in red. A detailed introduction to FE simulations and the geometry of the model are provided in section SIII of the Supporting Information. The calculated local lattice constant differs by 10–20 pm compared to the experimental values. In general, corrugation of the cleaved surface can alter the experimental profiles on the order of 50 pm;^53,54^ therefore, the 10–20 pm difference is within the acceptable error range. The difference might also arise from the approximations made for the FE simulations such as the QDs are square-based truncated pyramids, but in reality, the shape could deviate from this approximation, the cleaving position might be off-centered, etc. The peak values of both experimental and calculated lattice constant profiles match well within the acceptable error range. Even though the same analysis was not provided for layer A1, by comparing both X-STM topographic and current images, it is safe to assume that QDs in layer A1 are also nearly pure InAs in composition. Thus, by combining X-STM filled-state topographic, current images, and FE simulations, it is clear that the DEQDs in both layers A1 and A2 have pure InAs composition with tiny intermixing close to the QD edges.

### 2.4 Differences in Wetting Layer

**Figure 7:**
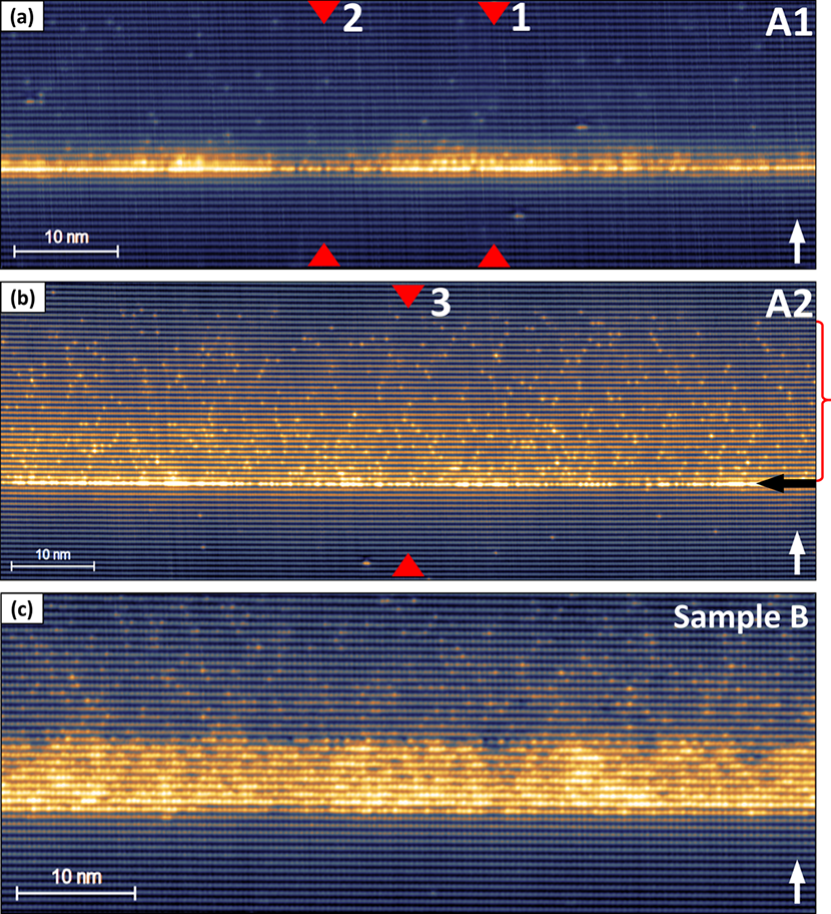
X-STM filled-state topographic images of the wetting layers in layers A1 (a) and A2 (b) in comparison with the WL from sample B (c) on top of a 5 nm InGaAs layer taken at *V*_b_ = −3.0 V and *I*_t_ = 50 pA. The red arrowheads (1, 2, and 3) indicate the positions where the STM height profiles are taken for Figure 8a. The growth direction [100] is indicated by the white arrows.

In conventional SK mode, the QD growth begins with the formation of a WL and, after a critical thickness is reached, the 3D islands are formed as a part of the strain relaxation of the WL. Therefore, the WL is unavoidable in SK growth while there is no such requirement in DE growth, yet we observed a partial WL or quasi-WL as shown in Figure 7. The growth surface is In-rich during the droplet deposition, and it is well-known that As replaces P at the growth front leading to the formation of a thin InAs(P) layer.^22,45^Figure 7a shows the topography of a thin quasi-WL in layer A1 where the QDs are crystallized at 480 °C; it is clear that the WL is a discontinuous layer of InAs(P) with As-rich (arrowhead 1) and As-poor (arrowhead 2) regions. Similar quasi-WL formation can be seen in Figure 7b for layer A2 where the QDs are crystallized at 520 °C. A high As concentration can be seen in the first 1–2 BLs (indicated with a black arrow in Figure 7b) due to the surface As–P exchange during the crystallization of the droplets. Additionally, a dilute InAs(P) layer with a thickness of almost 30 BLs (curly bracket in Figure 7b) formed due to the segregation of As from the WL into the capping layer as a result of higher crystallization temperature. This partial WL formation, i.e., the first 1–2 BLs in both layers A1 and A2, is comparable to the formation of indium-rich clusters during the growth of submonolayer QDs where the QDs are formed by stacking ML high islands.^55,56^ In the DE growth, the leftover indium on the growth surface from the droplet deposition reacts with the fresh As arriving on the surface forming such InAs-rich regions. In addition, the As–P surface exchange assists in the formation of a discontinuous layer of InAs(P). Holewa et al.^49^ suggested a 3 MLs thick InAs_0.5_P_0.5_ and 15 nm thick InAs_0.2_P_0.8_ cloud layer formed at 550 °C. As shown in Figure 7a, there is no such uniform InAs(P) cloud layer formation in layer A1, while in layer A2 (Figure 7b) and sample B (Figure 7c) we observe a nonuniform dilute InAs(P) layer formed purely due to As segregation with an exponential decay in As concentration. Apparently, the higher crystallization temperature used by Holewa et al.^49^ led to a thicker layer due to an increased temperature-driven As–P exchange reaction and strain-driven intermixing of the QD. The quasi-WL (first 1–2 BLs) is formed due to the As–P exchange, while the dilute InAs(P) layer is formed due to As segregation into the capping layer. The AsH_3_ fllux, substrate temperature, and crystallization time^34^ determine the thickness and composition of the dilute InAs(P) layer. In Figure 7c, we show the topography of the 5 nm thick InGaAs layer in sample B. It is difficult to differentiate the quasi-WL (first 1–2 BLs) on top of the InGaAs layer, but we observe a dilute InAs(P) layer in the capping region with a similar As concentration as in layer A2 due to the segregation of As atoms in the growth direction. The insertion of the InGaAs layer prior to the indium droplet deposition significantly reduces the As–P surface exchange and modifies the surface adatom mobility compared to InP, as also pointed out by Sala et al.^35^ Therefore, insertion of a thin InGaAs layer is an efficient method to suppress the substrate etching and reduce the surface As–P exchange in InAs/InP DEQDs.

**Figure 8:**
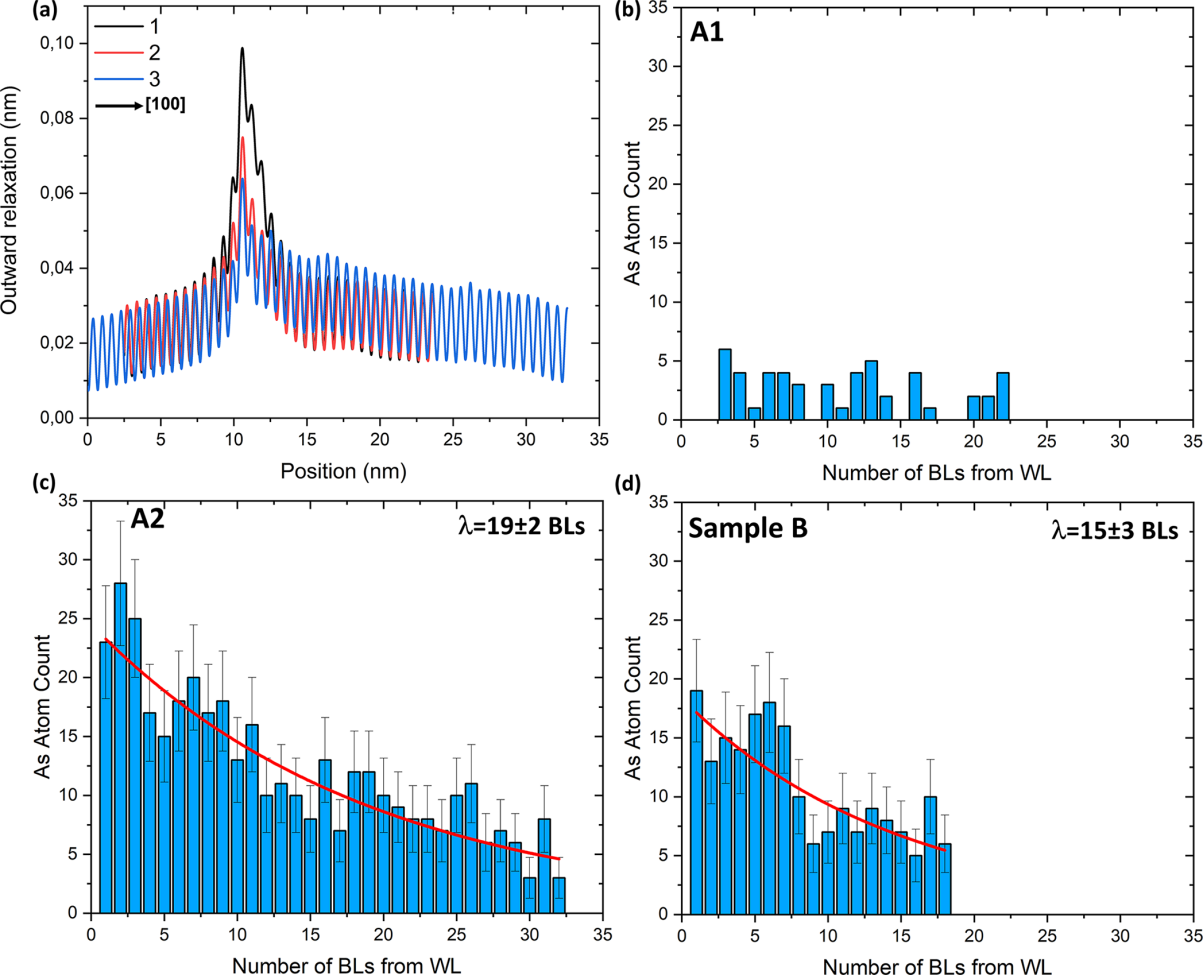
(a) STM height profiles of WLs in layer A1 (As-rich, 1; As-poor, 2) and layer A2 (3) taken along the growth direction [100]. The red arrowheads in Figure 7a,b indicate the area where the profiles are taken. The total numbers of As atoms counted in each BL from the WL into the capping layer for layer A1 (b), layer A2 (c), and sample B (d) are shown. (b) Leftover As from the crystallization step randomly incorporated into the capping layer of layer A1. (c) As segregation profile showing a clear exponential decay (red) due to the higher crystallization temperature can be seen in layer A2. (d) A similar As segregation profile can be seen in the capping layer of sample B showing an exponential decay (red) in the growth direction [100]. The exponential decay equation  was used to fit the segregation profile, where λ is the segregation length.

The compressively strained InAs(P) layers relax outward upon cleaving which can be measured from the X-STM height profiles. We show the X-STM height profiles in Figure 8a, where profile 1 (arrowhead 1 in Figure 7a) taken on the As-rich WL from layer A1 has the highest relaxation compared to profile 2 on the As-poor WL (arrowhead 2 in Figure 7a) and profile 3 on the WL in layer A2 (arrowhead 3 in Figure 7b). The effect of As segregation due to higher crystallization temperature can be clearly seen in STM height profile 3 (blue) from layer A2. The high-resolution images shown in Figure 7 are filled-state topographic images (group V sublattice) allowing us to count the individual As atoms in the capping layer using the atom counting method explained in section SIV of the Supporting Information. We note that the atom counting method is efficient only in the case of low concentrations as in the capping layer. At high concentrations, it is difficult to differentiate individual As atoms. Figure 8b shows the total number of As atoms in each BL from the WL into the capping layer in layer A1. The leftover As in the growth chamber from the previous crystallization step is randomly incorporated into the capping layer as seen in Figure 8b. The total number of As atoms in each BL from the WL in layer A2 is given in Figure 8c with an exponential decay fit (red). The exponential decay in the concentration is a typical signature of segregation. This segregation of As atoms is due to the increased crystallization temperature from layer A1 (480 °C) to layer A2 (520 °C). A similar segregation profile can be seen in sample B as shown in Figure 8d, where the crystallization temperature is the same as that for layer A2. It appears to be a little weaker than that in Figure 8c, possibly due to the fact that the total numbers of BLs counted are different for both layers. The composition of the dilute InAs(P) layer is higher in sample B, as the As concentration is averaged only for 18 BLs compared to 32 BLs in layer A2. By comparing parts b and c of Figure 8, it is evident that the segregation of As atoms from the WL into the capping layer is strongly dependent on the crystallization temperature in the InAs/InP DEQDs.

## 3 Conclusions

In summary, we presented a detailed atomic-resolution study of the morphology and substrate etching in the InAs/InP DEQDs grown by MOVPE using X-STM. The QDs crystallized at higher temperatures (layer A2) are much more size uniform than the QDs crystallized at a lower temperature (layer A1). Additionally, QDs in layer A2 show a perfect trapezoidal shape and are nearly InAs pure, suggesting a highly controllable MOVPE growth process. Two different etching processes were observed depending on the crystallization temperature: local drilling and long-range etching. The latter was reported here for the first time with atomic resolution. In local drilling, the droplet liquefies the InP underneath and the P atoms can easily diffuse out of the droplet to the edges. Later, during the crystallization step, the As atoms diffuse into the droplet and crystallize at the solid–liquid interface forming a nearly pure InAs etch pit underneath the QD. In long-range etching, due to the higher crystallization temperature, the surface InP layer is destabilized and the In atoms migrate into the droplet from the surroundings while the P atoms easily escape back into the growth chamber, forming trenches around the QD. We found that local drilling is dominant for crystallization temperatures of ≤500 °C, while long-range etching is dominant at temperatures of >500 °C. Interestingly, both etching processes can be suppressed by forming the droplets on a thin layer of InGaAs instead of InP. The presence of the InGaAs layer also significantly reduces the As–P surface exchange. Finally, we estimated the QD composition as pure InAs by performing FE simulations and fitting the experimental local lattice constant of the cleaved QD with the calculated lattice constant. Moreover, by comparing our results with the study of Holewa et al.^49^ on the same material system, we found that keeping the crystallization temperature below 540 °C prevented the incorporation of P atoms into the QDs, by suppressing As–P exchange reactions, thus leading to the formation of pure InAs QDs. We also compared the wetting layer formation on both InP and InGaAs layers, addressing the segregation of As atoms into the capping layer forming a dilute InAs(P) layer with an exponential decay in As concentration at 520 °C on both InP and InAs. Our atomic-resolution study sheds light on the morphology and etching behavior of various InAs/InP DEQDs grown by MOVPE. Thus, this work is a step forward in understanding the indium droplet crystallization mechanism at different temperatures, and it provides valuable feedback toward the large-scale fabrication of high-quality InAs/InP DEQDs at the telecom C-band for quantum information technologies.

## 4 Materials and Methods

Two samples were grown on an n-doped InP (100) substrate in a 3 × 2 close coupled showerhead (CCS) Aixtron metal–organic vapor phase epitaxy (MOVPE) reactor, using H_2_ as the carrier gas. Sample A has two layers of QDs, where the In droplets are formed at 320 °C followed by crystallization at 480 °C for layer A1 and at 520 °C for layer A2 in an As-rich environment. Both layers are separated by an 80 nm thick InP layer. In sample B, the In droplets are deposited on top of a 5 nm InGaAs layer at a temperature of 400 °C followed by crystallization at 480 and 520 °C to observe the effects of the InGaAs layer on droplet formation, etching, and evolution into a QD. Both samples are overgrown by 130 nm of InP to cap the QDs. The metal–organic precursor trimethylindium (TMIn) is used for In droplet deposition. Phosphine (PH_3_) and arsine (AsH_3_) were used to grow InP and droplet crystallization, respectively. An indium flow of 20 sccm was supplied for 35 s for indium droplet deposition in all the layers, and an arsenic flow of 0.5 sccm was maintained during the whole crystallization process. Detailed growth sequence, atomic force microscopy (AFM), and photoluminescence (PL) measurements on identical QDs were reported by Sala et al.^22,35,57^

The X-STM measurements were performed in a conventional Omicron low-temperature STM at 77 K under ultrahigh vacuum (UHV) with a base pressure of (4–6) × 10^–11^ mbar. The sample was cleaved in UHV to reveal one of the {110} natural cleaving planes of zincblende crystal. Polycrystalline tungsten wires were electrochemically etched to obtain STM tips followed by additional baking and Ar sputtering inside the STM preparation chamber in UHV. Detailed explanations of the X-STM technique and finite element simulations were already reported in previous publications.^12,33,34^

## Supporting Information Available

X-STM current images, X-STM images of QDs with 1 nm InGaAs interlayer, explanation of finite element modeling, and atom counting method (PDF)

## Notes

The raw data is available from the corresponding author upon reasonable request.
