# Supplementary material for: Control of Morphology and Substrate Etching in InAs/InP Droplet Epitaxy Quantum Dots for Single and Entangled Photon Emitters
Source: ACS Appl Nano Mater. 2022 May 30;5(6):8070–9. doi: 10.1021/acsanm.2c01197 (PMC9237823; doi:10.1021/acsanm.2c01197)
Supplement: Supplementary file 1 — an2c01197_si_001.pdf [file an2c01197_si_001.pdf]

Control of Morphology and Substrate Etching in InAs/InP Droplet Epitaxy Quantum Dots for Single and Entangled Photon Emitters

Raja Sekhar Reddy Gajjela[1,*], Elisa Maddalena Sala[2,3], Jon Heffernan[2,3], and Paul M. Koenraad[1]

[1]Department of Applied Physics, Eindhoven University of Technology, Eindhoven 5612 AZ, The Netherlands

[2]EPSRC National Epitaxy Facility, The University of Sheffield, North Campus, Broad Lane, S3 7HQ Sheffield, United Kingdom

[3]Department of Electronic and Electrical Engineering, The University of Sheffield, Sir Frederick Mappin Building, Mappin Street, S1 3JD Sheffield, United Kingdom

*r.s.r.gajjela@tue.nl

SI: X-STM current images

As mentioned in the manuscript, the current images are more sensitive to the local changes in composition. The alloy fluctuations can be easily identified in the X-STM current images.

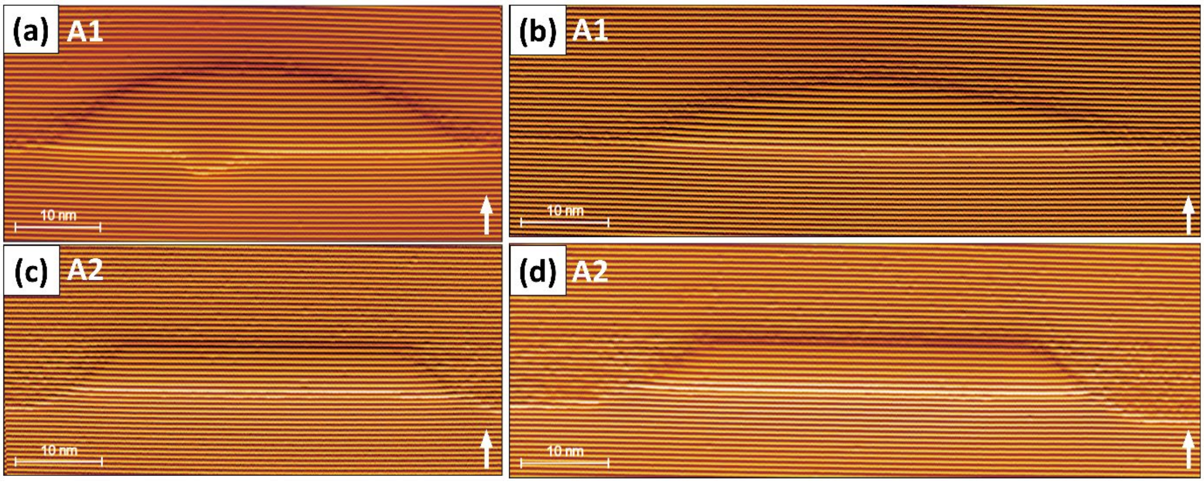

Figure.S 1: X-STM current images of the same DEQDs in layer A1 (a and b) and layer A2 (c and d) as shown in Figure.1 of the manuscript taken at the same tunneling conditions Vb = -3.0 V, It = 50 pA. The arrow indicates the growth direction [001].

The abrupt change in the current response due to compositional fluctuations and the suppressed topographic contrast makes it easy to identify alloy fluctuations within the QDs meaning that a pure QD (e.g. InAs) should give a uniform contrast in the current image. The point-like features close to the wetting layer are the segregated As atoms. We do not observe any such features inside the QD leading to the conclusion that the QDs are indeed pure without any alloying. The X-STM current images in conjunction with lattice constant measurement and finite element simulations can be used to derive the composition of the material under study.

SII: X-STM Images of QDs on InGaAs layer

As mentioned in the manuscript, growing a thin InGaAs layer suppresses both the etching effects and also the surface As-P exchange preventing the formation of the quasi wetting layer. In the manuscript we presented topographic images of QDs on a 5 nm InGaAs layer crystallized at 520°C here we show the results from additional experiments by changing the crystallization temperature and also the thickness of the InGaAs layer. A thin layer (1 nm) of InGaAs is sufficient to suppress the etching process at temperatures below and above 500°C.

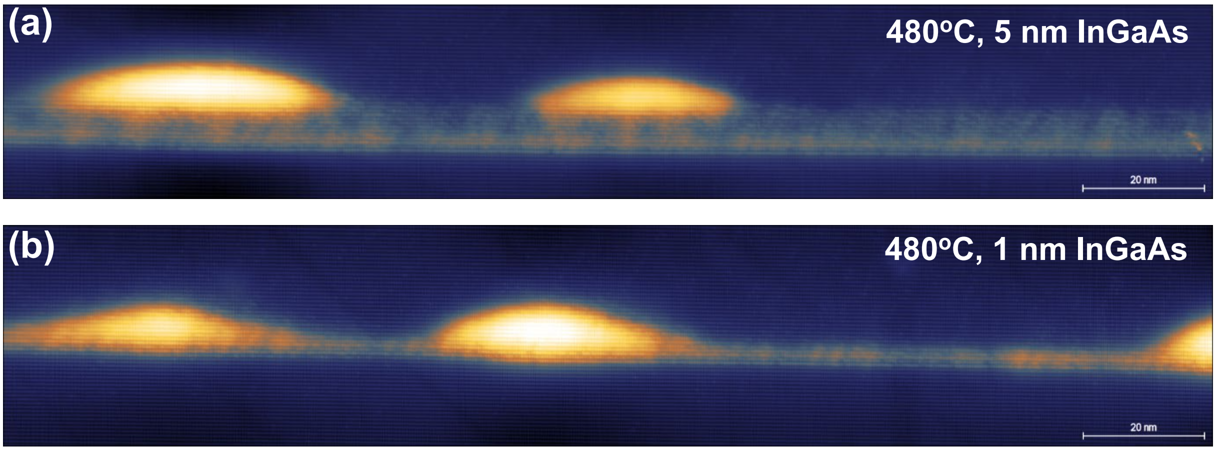

Figure.S 2: X-STM filled-state topographic images of DEQDs on 5 nm (a) and 1 nm (b) InGaAs layer crystallized at 480°C taken at V_b=-3.0 V and I_t=50 pA. Both local drilling and long-range etching mechanisms were suppressed even with a thin 1 nm InGaAs layer.

SIII: Finite Element Simulation

Simulations were performed with the finite element method (FEM) using the program: COMSOL Multiphysics to model the strain profile and surface relaxation of the quantum dots (QDs). COMSOL is a useful tool to simulate the outward relaxation and the local lattice constant using the solid-mechanics module. This can be applied in the determination of the material composition within a quantum well (QW) or quantum dot (QD) by comparing experimental and simulated results.

COMSOL numerically solves differential equations based on continuum elasticity theory. To start the simulation, the program needs a certain geometry of the QD or QW with the appropriate initial strain matrix. The initial strain will deform the cubic volume elements by acting as a force on their surfaces from different directions. The initial strain is caused by the lattice mismatch between the substrate material and the epitaxially grown layers. This lattice mismatch is given by:

$$\epsilon_0 = \frac{a - a_0}{a_0} \qquad \textit{Equation 1}$$

where a_0 is the original lattice constant of the substrate and a is the lattice constant of the epitaxially grown layers.

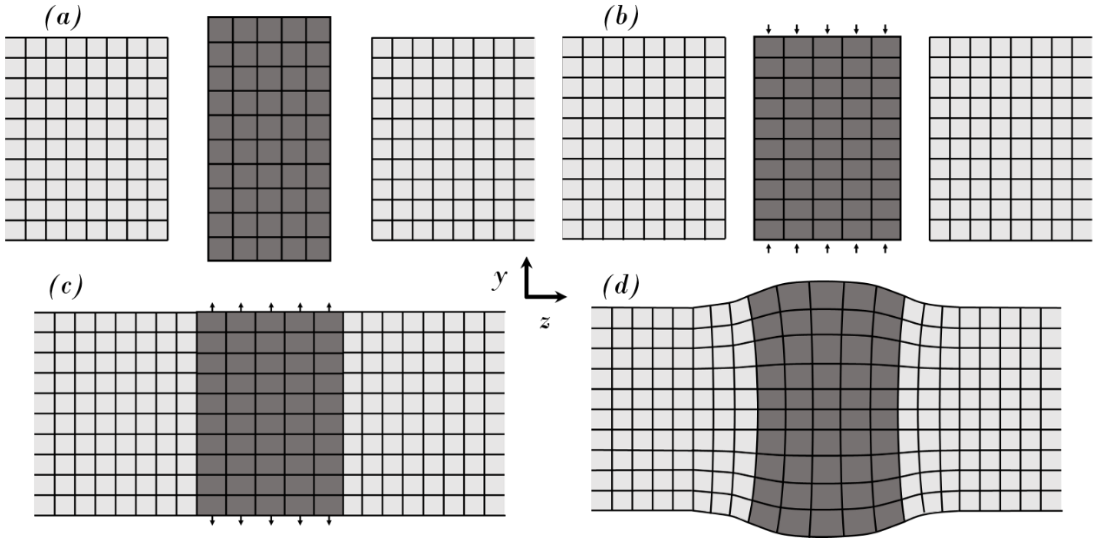

Figure.S 3: Schematic model of a strained quantum well at a surface: (a) Two materials with different lattice constants; (b and c) The QW has a higher lattice constant which is strained to be equal to the cladding; (d) outward relaxation of the cleaved QW.

In Figure.S 3, the four-step approach for a strained QW is shown: (a) QW and two cladding layers are brought close to each other. The QW's lattice constant exceeds the lattice constant of the cladding layers by the fraction ϵ_0 as defined by Equation 1. Here z is the growth direction, and y is the outward normal to the cleaved surface; (b) The lattice constant of the QW is now matched to the lattice constant of the cladding. Uniform stress in the x and y-direction decreases the lattice constant by ϵ_0. Due to the reduction of lattice constant in x and y, the lattice constant in the z-direction will increase as defined by the Poisson ratio $'\nu'$; (c) Now the QW is joined with the cladding layers without any additional strain. The initial strain of a QW is defined to be:

$$\epsilon_{QW} = \begin{bmatrix} \epsilon_{xx} & \epsilon_{xy} & \epsilon_{xz} \\ \epsilon_{yx} & \epsilon_{yy} & \epsilon_{yz} \\ \epsilon_{zx} & \epsilon_{zy} & \epsilon_{zz} \end{bmatrix} = \begin{bmatrix} -\epsilon_0 & 0 & 0 \\ 0 & -\epsilon_0 & 0 \\ 0 & 0 & \frac{2\nu\epsilon_0}{1-\nu} \end{bmatrix} \qquad \textit{Equation 2}$$

where ϵ_0 is the lattice mismatch, ν is the Poisson's ratio (which is a material property), and z is the growth direction[1,2]; (d) when the material is cleaved, outward pressure is applied to the exposed surface. This will displace the surface of both the cladding layers and the QW, which is the relaxation that can be experimentally measured with an X-STM.

For a QD, the approach is slightly different, as seen in Figure.S 4. This is due to the change in the shape of the structure. Instead of a full slab, only a small volume with limited dimensions has to be fitted inside the cladding material. Therefore, the lattice constant of the full QD is reduced equally in all three dimensions (x,y,z) by the lattice mismatch to match the lattice constant of the cladding. In matrix form this is written as:

$$\epsilon_{QW} = \begin{bmatrix} \epsilon_{xx} & \epsilon_{xy} & \epsilon_{xz} \\ \epsilon_{yx} & \epsilon_{yy} & \epsilon_{yz} \\ \epsilon_{zx} & \epsilon_{zy} & \epsilon_{zz} \end{bmatrix} = \begin{bmatrix} -\epsilon_0 & 0 & 0 \\ 0 & -\epsilon_0 & 0 \\ 0 & 0 & -\epsilon_0 \end{bmatrix} \qquad \textit{Equation 3}$$

The elastic properties are described with 3 independent values (C_{11}, C_{12}, and C_{44}) in a simple cubic-symmetric case given by the stiffness matrix (D):

$$D = \begin{bmatrix} C_{11} & C_{12} & C_{12} & 0 & 0 & 0 \\ C_{12} & C_{11} & C_{12} & 0 & 0 & 0 \\ C_{12} & C_{12} & C_{11} & 0 & 0 & 0 \\ 0 & 0 & 0 & C_{44} & 0 & 0 \\ 0 & 0 & 0 & 0 & C_{44} & 0 \\ 0 & 0 & 0 & 0 & 0 & C_{44} \end{bmatrix} \qquad \textit{Equation 4}$$

This elasticity matrix is then used in the stress-strain relation as:

$$\sigma = D\,\epsilon \qquad \textit{Equation 5}$$

where the stress and strain are 6×1 vectors:

$$\sigma = \begin{bmatrix} \sigma_{xx} \\ \sigma_{yy} \\ \sigma_{zz} \\ \sigma_{xy} \\ \sigma_{yz} \\ \sigma_{zx} \end{bmatrix}, \epsilon = \begin{bmatrix} \epsilon_{xx} \\ \epsilon_{yy} \\ \epsilon_{zz} \\ \epsilon_{xy} \\ \epsilon_{yz} \\ \epsilon_{zx} \end{bmatrix} \qquad \textit{Equation 6}$$

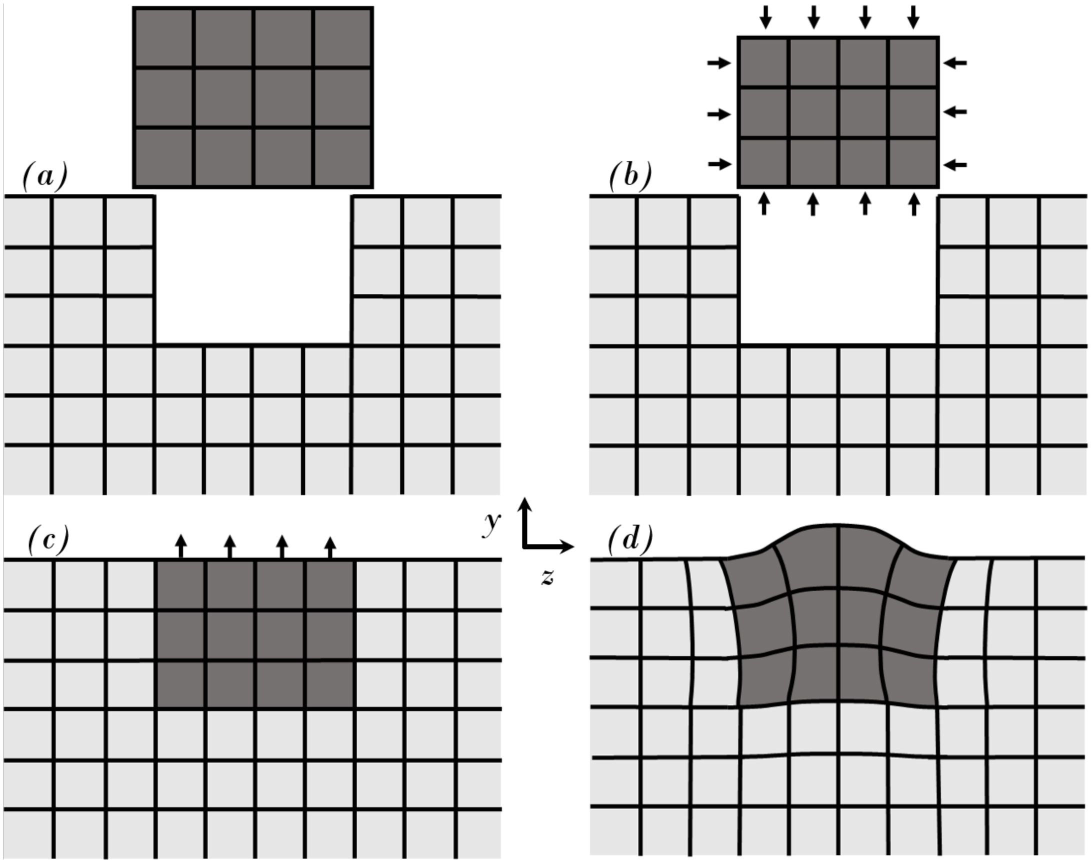

Figure.S 4:Schematic model of a strained quantum dot at a surface: (a) Two materials with different lattice constants; (b and c) The higher lattice constant of the QD is strained equally in all three dimensions to fit the cladding; (d) Outward relaxation of the cleaved QD.

Some critical steps need to be followed to obtain an accurate result during the FE simulation. First, a large enough box (of cladding material) should be placed around the simulated structure to eliminate non-physical interactions from the boundaries. Second, appropriate boundary conditions should be applied to avoid any error in the simulation. The front side (which will relax outwards) should be set free, the backside is fixed, and the other four surfaces have symmetric or periodic boundary conditions: so, their movement is restricted within their own plane. Third, the initial strain matrix is applied which depends on the shape of the earlier discussed nanostructures (the initial strain matrix varies from QW to QD). In a multi-layered system with various materials, the grown layers are always strained to the substrate material to match the lattice constants. Fourth, a fine enough mesh should be generated to have high accuracy in the simulation. Finally, COMSOL calculates the equilibrium position and the final strain values. The vertical displacement component of the simulation can be compared with the outward relaxation of the cleaved surface. The local lattice constant $a_{calc}(z)$ is calculated from the final strain $\epsilon_{zz,calc}$ and initial lattice constant $a_{ini}(z)$ as follows:

$$a_{calc}(z) = \left(\epsilon_{zz,\,calc} + 1\right) \times a_{ini}(z) \qquad \textit{Equation 7}$$

which can then be compared to the local lattice constant measurements obtained from X-STM experiments.

The main steps for creating a FEM simulation on a cleaved QD are as follows: create a geometry, apply the correct boundary conditions, input the initial strain conditions, and render the mesh. The geometry and mesh can be seen in Figure.S 5. From the X-STM analysis, we know that the QDs have a truncated pyramid shape and the cleaving is parallel to the diagonal of the square base pyramid. As mentioned in the manuscript, DEQDs have a discontinuous wetting layer, for the sake of simplicity, no wetting layer is simulated in FEM, as highlighted in Figure.S 5. The material composition is pure InAs for QD capped with InP. The dimensions of the InP block are large enough to avoid any boundary effects (100 nm). The two planes in the x-direction have symmetric boundary conditions which make the system act as if it were repeated infinitely along the x-direction. The top and bottom planes (z-direction) were also made with symmetric conditions to negate boundary effects. The backside (negative y-direction) is fixed, and the front side (positive y-direction) is free, so the surface can relax outwards in that direction when “cleaved”. For the relaxation to take place an initial strain is needed. Since we do not have a wetting layer we can use QD strain to provide the initial strain condition (Equation 3). The program can now calculate the equilibrium positions for the relaxation of the surface and the final strain values under stationary boundary conditions. The lattice constant was calculated according to Equation 7.

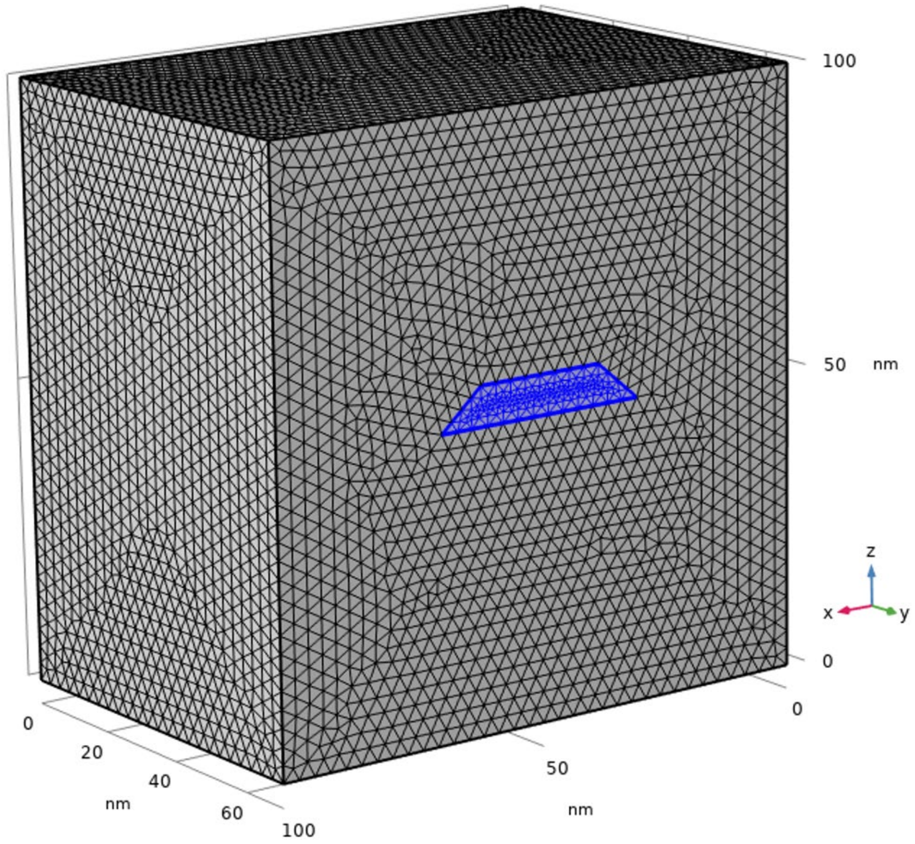

Figure.S 5:Geometry and mesh of the COMSOL model used to perform FE simulation. Here, z is the growth direction and the positive y direction is normal to the cleaving plane. The most important region highlighted in blue is the InAs QD.

SIV: Atom counting method

A filled-state X-STM imaging group V sublattice (P and As atoms) is shown with a line profile along the [100]. The presence of As atoms in the capping layer shows a different height in the STM height profile compared to the background P atoms as shown in Figure.S 6 where 5 individual peaks can be identified due to the presence of As atoms randomly incorporated into the capping layer.

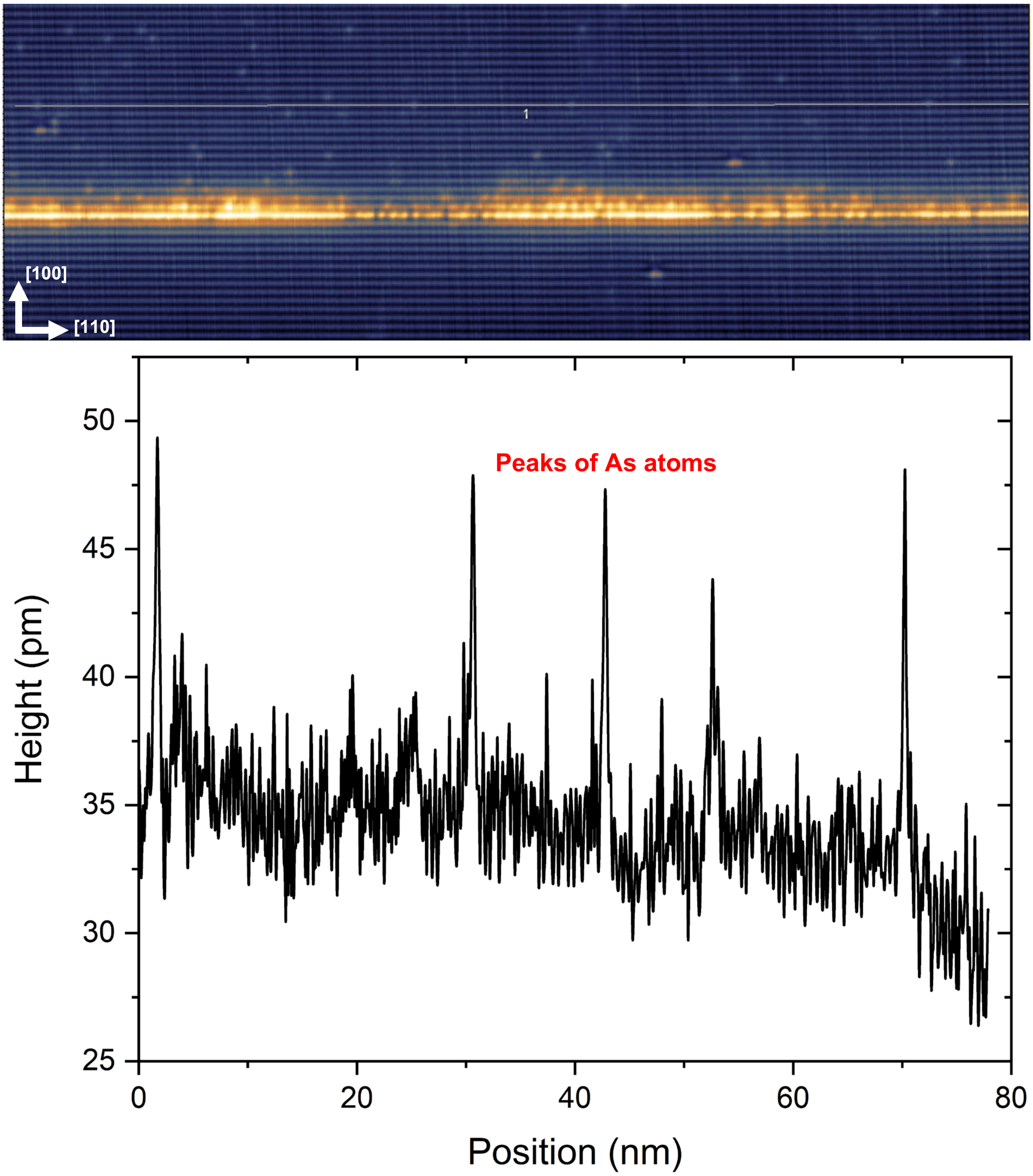

Figure.S 6:X-STM filled-state image (group V sublattice) is shown with a line profile along [110] direction showing the peaks due to surface and subsurface As atoms randomly incorporated into the capping layer.

Manual counting combined with peak identification using STM line profiles can be used to count the number of As atoms present in each corrugation line and to estimate the concentration of As on each corrugation line in the capping layer. The brightness of the feature is directly proportional to the distance from the surface, atoms on the surface being the brightest. The line profile taken along the [110] direction given in Figure.S 6 shows the height of the bright features relative to the surface. Note that if the concentration goes beyond 10-15% as in the wetting layer (WL) region, it is nearly impossible to differentiate one atom from another. Therefore we counted the As atoms in all layers except the WL region. In this way, the maximum number of As atoms for a given size can be measured, and dividing the same with the available number of lattice sites provides the local As concentration.

References:

(1) Davies, J. H.; Bruls, D. M.; Vugs, J. W. A. M.; Koenraad, P. M. Relaxation of a Strained Quantum Well at a Cleaved Surface. *J. Appl. Phys.* **2002**, *91* (7), 4171–4176. https://doi.org/10.1063/1.1459100.

(2) Davies, J. H.; Offermans, P.; Koenraad, P. M. Relaxation of a Strained Quantum Well at a Cleaved Surface. Part II: Effect of Cubic Symmetry. *J. Appl. Phys.* **2005**, *98* (5), 053504. https://doi.org/10.1063/1.2030415.
